# Recent breast cancer trends among Asian/Pacific Islander, Hispanic, and African-American women in the US: changes by tumor subtype

**DOI:** 10.1186/bcr1839

**Published:** 2007-12-27

**Authors:** Amelia K Hausauer, Theresa HM Keegan, Ellen T Chang, Christina A Clarke

**Affiliations:** 1 Northern California Cancer Center, 2201 Walnut Avenue, Suite 300, Fremont, CA 94538, USA; 2 Division of Epidemiology, Department of Health Research and Policy, Stanford School of Medicine, Stanford University, 259 Campus Drive, Stanford, CA 94305, USA

## Abstract

**Background:**

Recently, unprecedented drops in breast cancer incidence have been reported for populations of mostly White European descent. Incidence patterns in non-White racial/ethnic groups are less described. Therefore, we examined population-based breast cancer incidence trends separately for US Asian/Pacific Islander, Hispanic, African-American, and non-Hispanic White women by etiologically relevant tumor subtype characteristics, including hormone receptor status, histology, size, and *in situ *behavior.

**Methods:**

We obtained breast cancer data from 13 Surveillance, Epidemiology, and End Results (SEER) cancer registries to calculate age-adjusted incidence rates and trends, stratified by race/ethnicity and tumor subtype for the period 1992–2004. Detailed analyses were limited to women 50 years old or older. Joinpoint regression was used to assess incidence trends by annual quarter of diagnosis.

**Results:**

Between 2001 and 2004, incidence rates of invasive breast cancer in women 50 years old or older declined appreciably among Asians/Pacific Islanders (-8.5%) and Hispanics (-2.9%) and were stable in African-Americans (+0.5%), reductions substantially lower than those observed among non-Hispanic Whites (-14.3%). In Asian/Pacific Islander women, perceptible but statistically nonsignificant decreases were observed for hormone receptor-positive, lobular, and small tumors only. Rates of hormone receptor-negative tumors increased among African-Americans (26.1%) and Hispanics (26.9%) during 2001–2004. Incidence trends in most groups, except African-American women, peaked between 1999 and mid-2002. Rates of *in situ *cancer remained stable in all groups.

**Conclusion:**

Recently reported reductions in breast cancer incidence varied considerably by race/ethnicity. These patterns are consistent with documented racial/ethnic differences in the prevalence and discontinuation of hormone therapy (HT) after July 2002 but do not correspond as well to patterns of mammography use in these groups. The data presented in this analysis provide further evidence that population-level HT use is a major influence on population-level rates of particular breast cancer subtypes, especially receptor-positive tumors.

## Introduction

Recent reports have documented sudden, unprecedented declines in the incidence of breast cancer, particularly for invasive, estrogen receptor-positive (ER^+^) tumors diagnosed in women 50 years old or older [1-7]. Thus far, substantial drops have been observed in the US, Germany, New Zealand, and Canada [1-3,8-10] but not in the Netherlands, Norway, or Sweden [10]. In populations reporting a decrease, gradual incidence declines began as early as 1999 but accelerated in 2002 after the early and widely publicized termination of the Women's Health Initiative (WHI) estrogen/progestin arm, in which the experimental group experienced increased risks of breast cancer [11]. The US incidence reductions generally have been attributed to two factors: (a) the well-documented mass cessation of menopausal hormone therapy (HT) beginning in the second half of 2002 [1-3,12-16] and (b) the possible effects of saturation in mammographic screening programs [2,5]. However, it is difficult to quantify precisely the relative impacts of these phenomena on breast cancer incidence because the US does not employ a comprehensive health tracking resource and must therefore rely on ecologic assessments for understanding population cancer patterns.

To date, recent incidence reductions have been well characterized for populations of mostly or entirely European descent (for example, non-Hispanic White women) but remain incomplete for populations of other races/ethnicities, especially by tumor subtype for which incidence patterns vary considerably, possibly because of etiologic heterogeneity [17-21]. One report did suggest that overall age-adjusted invasive breast cancer rates in US African-American women were essentially unchanged between 2001 and 2004 [22]. To better understand whether the recent incidence drops observed in non-Hispanic White women were also observed in women of non-White races/ethnicities, we examined trends in invasive and *in situ *female breast cancer by tumor hormone receptor status, histology, and size among US Asian/Pacific Islander, Hispanic, and African-American women as compared with non-Hispanic White women.

## Materials and methods

We obtained population-based breast cancer (*International Classification of Diseases for Oncology, 3rd Edition *[ICD-O-3], sites 50.0 to 50.9) incidence data from the Surveillance, Epidemiology, and End Results (SEER) program of the National Cancer Institute (Bethesda, MD, USA), including 321,157 cases of invasive and 66,074 cases of *in situ *disease diagnosed between 1992 and 2004 in SEER-13 catchment regions (Alaska natives; Connecticut; Hawaii; Iowa; New Mexico; rural Georgia; Utah; the metropolitan areas surrounding Atlanta, GA; Detroit, MI; Los Angeles, CA; San Francisco-Oakland, CA; San Jose-Monterey, CA; and Seattle-Puget Sound, WA). Altogether, the population covered by these 13 registries comprises 14% of the entire US population and is representative of the larger population with respect to educational and socioeconomic status but over-represents urban areas and foreign-born populations [23]. Demographic and tumor information for each incident case of breast cancer was abstracted directly from medical records [24]. Population denominator estimates were obtained from the SEER program and based on US census data.

Incidence analyses included all diagnoses reported in women between the years 1992, the first year that data from the large and diverse Los Angeles and San Jose-Monterey SEER regions were available, and 2004, the most recent year that SEER data were available. All analyses were limited to women more than 50 years old because (a) post-2002 incidence declines were not observed in the 5-year age groups under age 50 (data not shown); (b) women under age 50 are more heterogeneous with respect to menopausal status, HT use, and regularity of mammographic screening; and (c) breast cancer risk factors (for example, familial risk) may differ for premenopausal women. We examined rates and trends for invasive and *in situ *tumors separately, then stratified incidence by several demographic and tumor characteristics, including race (White, Black, or Asian/Pacific Islander), Hispanic origin, ER and progesterone receptor (PR) status (ER^+^/PR^+^, ER^+^/PR^-^, ER^-^/PR^+^, ER^-^/PR^-^, or other/unknown), histology (ICD-O-3 histology codes for invasive ductal carcinoma [IDC] [code 8500], invasive lobular carcinoma [ILC] [code 8520], invasive ducto-lobular carcinoma [IDLC] [code 8522], or other/unknown [codes 8000–8499, 8501–8519, 8521, and 8523–9989]), and tumor size (diameter of largest focus less than 2 cm, greater than or equal to 2 cm, or unknown). We categorized race/ethnicity into the following groups: Asian/Pacific Islander, Hispanic, African-American, and non-Hispanic White (hereafter referred to as White). Incidence rates were not subjected to modeling for possible delays in reporting, since prior analyses found that such adjustments had no substantial impact on breast cancer trends or patterns [2].

We used SEER*Stat version 6.3.5 (National Cancer Institute) to calculate annual age-adjusted breast cancer incidence rates and corresponding 95% confidence intervals (CIs). All rates are presented as cases per 100,000 person-years unless otherwise noted. Statistical significance between yearly rates was determined by comparing the CI overlap. The Joinpoint Regression Program version 3.0 (National Cancer Institute) was used to describe changes in quarterly incidence trends within tumor subtypes by racial/ethnic group. Joinpoint software fits a linear regression function to the data and then determines between zero and three joinpoints, indicating significant changes in the overall trend (significance level, *P *< 0.05). These significance tests use a Monte Carlo permutation method [25]. All data were plotted on a semilogarithmic scale to aid visual assessment of slope differences [26].

## Results

For the period 1992–2004, nearly 244,000 cases of invasive and 48,000 cases of *in situ *breast cancer in women over 50 years of age were reported to the SEER program. Table 1 outlines the demographic characteristics of these patients and clinical characteristics of their tumors stratified by race/ethnicity. Most of these tumors (79.3% of invasive and 77.1% of *in situ*) occurred in White women. Regardless of patient race/ethnicity, the majority of tumors were ER^+^/PR^+^, ductal histology, and small (diameter of less than or equal to 2 cm), except for among African-Americans, who were diagnosed with large tumors more frequently than small tumors. Both Hispanic and African-American women were more likely than Whites to present with large or hormone receptor-negative tumors, and Asians/Pacific Islanders were more likely than other racial/ethnic groups to have ductal rather than lobular or mixed tumors.

**Table 1:** Demographic and tumor characteristics of invasive and *in situ *female breast cancer cases (Surveillance, Epidemiology, and End Results-13)

	Invasive			
	Asian/Pacific Islander	Hispanic	African-American	Non-Hispanic White
	*n *= 15,933 (6.5)	*n *= 15,355 (6.3)	*n *= 19,105 (7.8)	*n *= 193,513 (79.3)
Demographics				
Diagnosis year				
1992	854 (5.4)	920 (6.0)	1,237 (6.5)	13,822 (7.1)
1993	851 (5.3)	869 (5.7)	1,231 (6.4)	13,732 (7.1)
1994	870 (5.5)	958 (6.2)	1,299 (6.8)	14,003 (7.2)
1995	984 (6.2)	995 (6.5)	1,356 (7.1)	14,333 (7.4)
1996	1,045 (6.6)	1,095 (7.1)	1,392 (7.3)	14,402 (7.4)
1997	1,253 (7.9)	1,067 (6.9)	1,453 (7.6)	15,289 (7.9)
1998	1,292 (8.1)	1,212 (7.9)	1,483 (7.8)	15,807 (8.2)
1999	1,373 (8.6)	1,264 (8.2)	1,550 (8.1)	16,048 (8.3)
2000	1,352 (8.5)	1,350 (8.8)	1,506 (7.9)	15,608 (8.1)
2001	1,491 (9.4)	1,327 (8.6)	1,526 (8.0)	16,033 (8.3)
2002	1,558 (9.8)	1,433 (9.3)	1,683 (8.8)	15,697 (8.1)
Tumor characteristics				
Hormone receptor status				
ER^+^/PR^+^	8,242 (51.8)	6,619 (43.1)	6,941 (36.3)	101,364 (52.4)
ER^+^/PR^-^	1,707 (10.7)	1,558 (10.1)	1,921 (10.1)	21,653 (11.1)
ER^-^/PR^+^	324 (2.0)	250 (1.6)	374 (2.0)	3,022 (1.6)
ER^-^/PR^-^	2,504 (15.7)	2,417 (15.7)	4,243 (22.2)	24,355 (12.6)
Other/unknown	3,156 (19.8)	4,511 (29.4)	5,626 (29.4)	43,119 (22.3)
Histology				
IDC	11,780 (73.9)	10,204 (66.5)	12,824 (67.1)	128,393 (66.3)
ILC	748 (4.7)	1,121 (7.3)	1,190 (6.2)	18,663 (9.7)
IDLC	843 (5.3)	1,152 (7.5)	1,179 (6.2)	14,963 (7.7)
Other/unknown	2,562 (16.1)	2,878 (18.7)	3,912 (20.5)	31,494 (16.3)
Size				
<2 cm	8,669 (54.4)	7,077 (46.1)	7,904 (41.4)	108,527 (56.1)
≥ 2 cm	6,076 (38.1)	6,674 (43.5)	8,711 (45.6)	66,515 (34.4)
Unknown	1,188 (7.5)	1,604 (10.4)	2,490 (13.0)	18,471 (9.5)
				
	*In situ*			
	Asian/Pacific Islander	Hispanic	African-American	Non-Hispanic White
	*n *= 4,025 (8.4)	*n *= 2,807 (5.9)	*n *= 4,072 (8.5)	*n *= 36,908 (77.1)
				
Demographics				
Diagnosis year				
1992	115 (2.9)	110 (3.9)	177 (4.3)	1,942 (5.3)
1993	163 (4.0)	117 (4.2)	181(4.4)	1,932 (5.2)
1994	173 (4.3)	138 (4.9)	212 (5.2)	2,043 (5.5)
1995	214 (5.3)	152 (5.4)	259 (6.4)	2,313 (6.3)
1996	219 (5.4)	160 (5.7)	238 (5.8)	2,411 (6.5)
1997	245 (6.1)	199 (7.1)	300 (7.4)	2,717 (7.4)
1998	292 (7.3)	237 (8.4)	335 (8.2)	3,182 (8.6)
1999	381 (9.5)	238 (8.5)	350 (8.6)	3,306 (9.0)
2000	358 (8.9)	257 (9.2)	383 (9.4)	3,344 (9.1)
2001	447 (11.1)	279 (9.9)	412 (10.1)	3,496 (9.5)
2002	527 (13.1)	287 (10.2)	375 (9.3)	3,497 (9.5)
2003	451 (11.2)	309 (11.0)	413 (10.1)	3,326 (9.0)
2004	440 (10.9)	324 (11.5)	437 (10.7)	3,399 (9.2)

Values in parentheses are percentages. ER, estrogen receptor; IDC, invasive ductal carcinoma; IDLC, invasive ducto-lobular carcinoma; ILC, invasive lobular carcinoma; PR, progesterone receptor.

### Trends for invasive cancer

**Figure 1:**
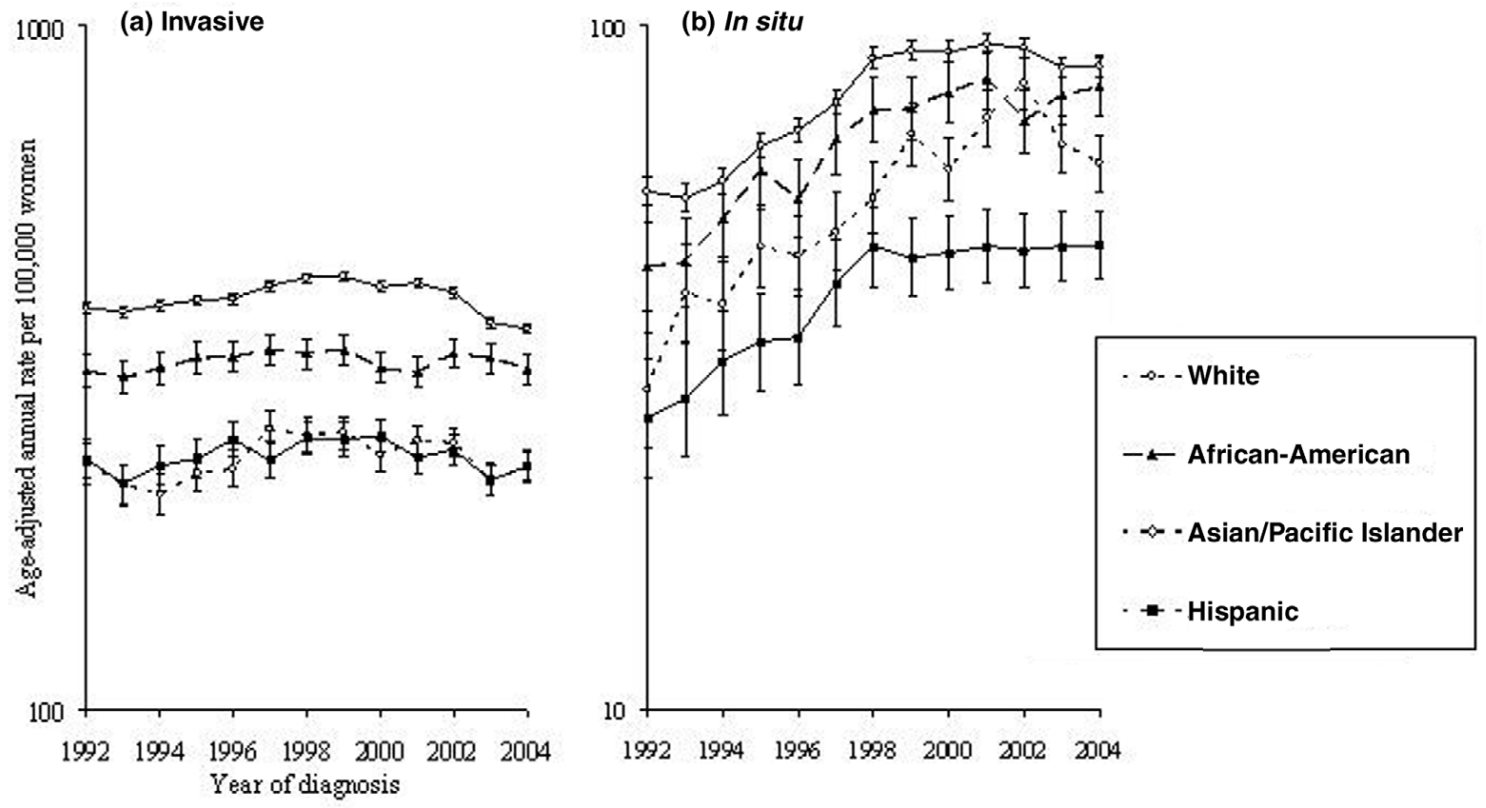
Breast cancer incidence among women 50 years old or older by tumor behavior, race/ethnicity, and year according to Surveillance, Epidemiology, and End Results-13. **(a) **Trends for invasive breast cancer. **(b) **Trends for *in situ *breast cancer. All rates are age-adjusted to the 2000 US standard.

Figure 1a shows that Asian/Pacific Islander (-8.5%) and Hispanic (-2.9%) women experienced statistically nonsignificant incidence declines between 2001 and 2004 (247.3, 95% CI 234.8 to 260.3 in 2001 and 226.4, 95% CI 215.2 to 237.9 in 2004 for Asians/Pacific Islanders; 233.8, 95% CI 221.2 to 247.1 in 2001 and 227.0, 95% CI 215.4 to 239.1 in 2004 for Hispanics). Rates among African-Americans were relatively stable (0.5%) over the same period (312.0, 95% CI 296.4 to 328.3 in 2001 and 313.7, 95% CI 298.7 to 329.3 in 2004). These results contrasted with the substantial decline observed among White women, in whom incidence fell from 421.3 (95% CI 414.7 to 427.9) in 2001 to 360.9 (95% CI 354.9 to 366.9) in 2004, a 14.3% decline.

Joinpoint regression analysis of invasive breast cancer by annual quarter of diagnosis suggested an incidence increase among White women until the first quarter of 1999 (0.5% per quarter, 95% CI 0.4% to 0.7%), stable rates from 1999 to 2002 (-0.4% per quarter, 95% CI -1.1% to 0.3%), and a significant decline thereafter (-1.4% per quarter, 95% CI -1.8% to -0.9%). By comparison, incidence in Asians/Pacific Islanders increased more dramatically from the third quarter of 1994 to the third quarter of 1997 (2.0% per quarter, 95% CI 0.7% to 3.4%) then declined more gradually (-0.5% per quarter, 95% CI -0.8% to -0.3%) through 2004. In Hispanics, rates increased by 0.4% (95% CI 0.1% to 0.7%) per quarter prior to 2000 and decreased by 0.7% (95% CI -1.3% to -0.2%) per quarter after 2000. Significant changes in incidence were not detected among African-American women between 1992 and 2004 (-0.02% per quarter, 95% CI -0.1% to 0.1%).

### Trends for *in situ *cancer

Incidence of *in situ *tumors remained constant during 2001–2004 among Asian/Pacific Islander, Hispanic, and African-American women (Figure 1b). In White women, rates decreased 7.0% from 94.2 (95% CI 91.1 to 97.4) in 2001 to 87.2 (95% CI 84.2 to 90.2) in 2004. Joinpoint analyses suggested that rates of *in situ *cancer increased during the first half of the observation period, irrespective of race/ethnicity, and then decreased in the first quarter of 2002 among Asians/Pacific Islanders (-3.3% per quarter, 95% CI -5.5% to -1.0%), a decline that began 3 years after the decreasing trend observed among Whites (-0.5% per quarter, 95% CI -0.8% to -0.1% after third quarter 1999). In contrast, rates for Hispanic and African-American women stabilized during 1998–1999 and were constant through 2004.

### Trends by tumor hormone receptor status

**Figure 2:**
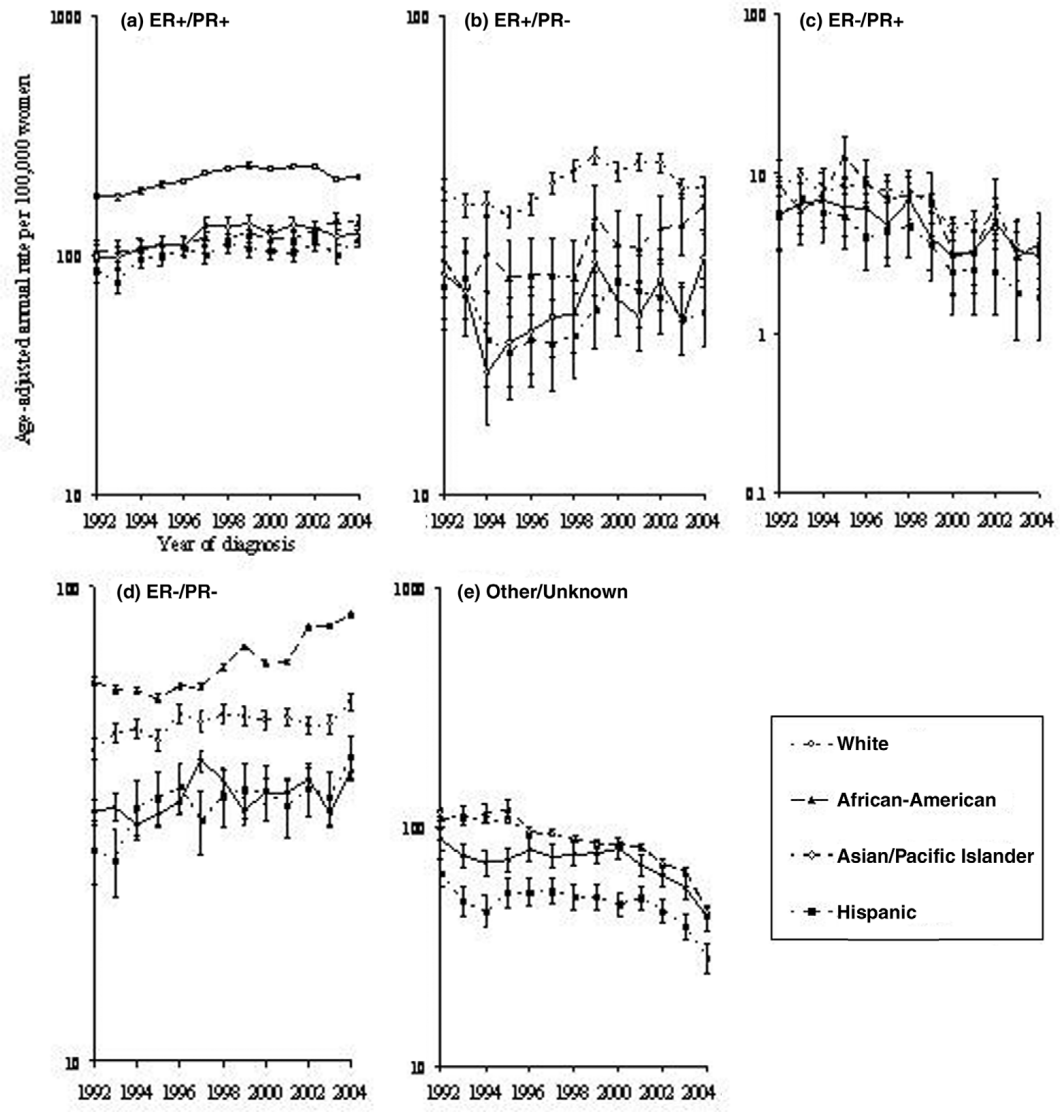
Invasive breast cancer incidence among women 50 years old or older by receptor status, race/ethnicity, and year according to Surveillance, Epidemiology, and End Results-13. **(a) **Trends for ER^+^/PR^+ ^breast cancer. **(b) **Trends for ER^+^/PR^- ^breast cancer. **(c) **Trends for ER^-^/PR^+ ^breast cancer. **(d) **Trends for ER^-^/PR^- ^breast cancer. **(e) **Trends for other/unknown breast cancer. All rates are age-adjusted to the 2000 US standard. ER, estrogen receptor; PR, progesterone receptor.

Figure 2 shows annual changes in hormone receptor-defined breast cancer rates among women 50 years old or older by race/ethnicity. Asian/Pacific Islander women experienced a perceptible yet nonsignificant decrease (though more attenuated than the significant 8.6% decline observed in White women) in the rate of ER^+^/PR^+ ^tumors, producing a cumulative reduction of 7.6% between 2001 and 2004. An increase in the incidence rate of ER^-^/PR^- ^tumors occurred among Hispanic women, in whom rates rose 26.8% from 34.6 (95% CI 29.9 to 39.9) to 43.9 (95% CI 39.0 to 49.9) during the period 2001–2004, and among African-American women, in whom rates rose 26.1% from 69.3 (95% CI 62.1 to 77.0) to 87.4 (95% CI 79.6 to 95.8). ER^-^/PR^+ ^tumors were uncommon but appeared to decline consistently over the time period.

Distinctly increasing and decreasing trends in ER^+^/PR^+ ^tumors were evident in Asians/Pacific Islanders (1.5% per quarter, 95% CI 1.1% to 2.0% until the first quarter of 1998; -0.3% per quarter, 95% CI -0.7% to -0.001%) but were not detectable in Hispanics or African-Americans as compared with trends in Whites (1.3% per quarter, 95% CI 1.1% to 1.5% from mid-1992 to mid-1999; -0.7%, 95% CI -0.9% to -0.4% per quarter thereafter). Increases in the incidence of ER^-^/PR^- ^tumors between 1992 and 2004 were observed for African-American and Hispanic women only.

### Trends by histologic subtype

**Figure 3:**
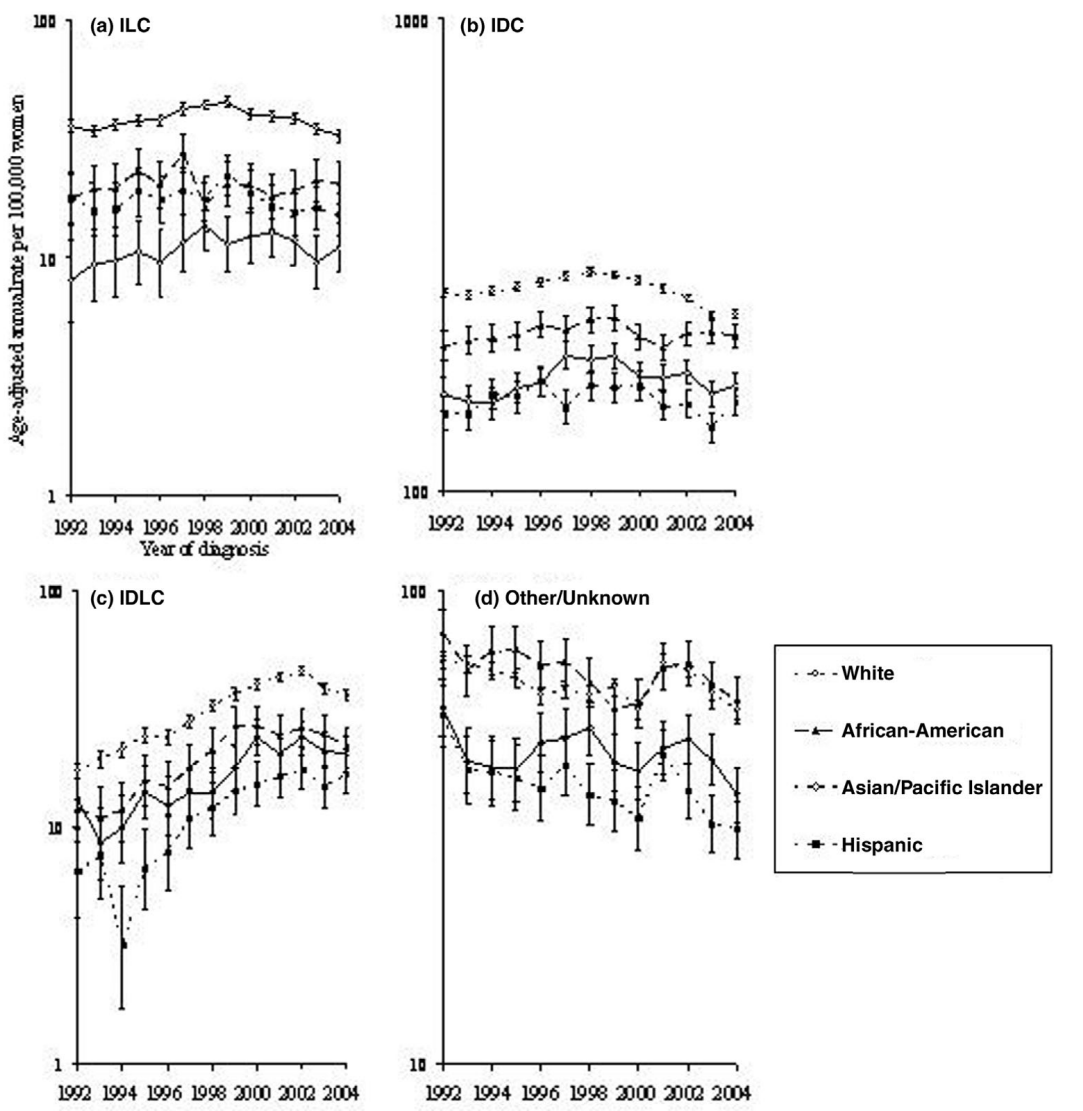
Invasive breast cancer incidence among women 50 years old or older by tumor histology, race/ethnicity, and year according to Surveillance, Epidemiology, and End Results-13. **(a) **Trends for ILC breast cancer. **(b) **Trends for IDC breast cancer. **(c) **Trends for IDLC breast cancer. **(d) **Trends for other/unknown breast cancer. All rates are age-adjusted to the 2000 US standard. IDC, invasive ductal carcinoma; IDLC, invasive ducto-lobular carcinoma; ILC, invasive lobular carcinoma.

Incidence rates for specific histologic subtypes did not change appreciably between 2001 and 2004 in any racial/ethnic group except for Whites, in whom ILC dropped 17.3%, IDLC 16.6%, and IDC 12.0% (Figure 3). Notably, due to the small number of cases of ILC and IDLC breast cancer among Asians/Pacific Islanders, Hispanics, and African-Americans, rates were considerably more variable in minority groups than in Whites.

Using Joinpoint regression, we found two distinct patterns for ILC in Asian/Pacific Islander women: a statistically significant increase from 1992 to mid-1998 (1.8% per quarter, 95% CI 0.5% to 3.2%) followed by a nonsignificant decrease from mid-1998 to 2004 (-0.9% per quarter, 95% CI -2.0% to 0.2%). Trends for IDC were similar but more moderate. The incidence rate of IDLC increased significantly in Hispanic and Asian/Pacific Islander women across the entire observation period (1.6% per quarter, 95% CI 1.2% to 2.1%, and 2.1% per quarter, 95% CI 1.5% to 2.7%, respectively) and in African-Americans from 1992 to 2000 (3.4% per quarter, 95% CI 2.6% to 4.2%), after which rates stabilized (-1.1% per quarter, 95% CI -2.4% to 0.2%). Conversely, IDLC among White women rose by 2.6% (95% CI 2.4% to 2.8%) per quarter prior to 2002 then rapidly declined at -2.8% (95% CI -3.7% to -1.9%) per quarter.

### Trends by tumor size

**Figure 4:**
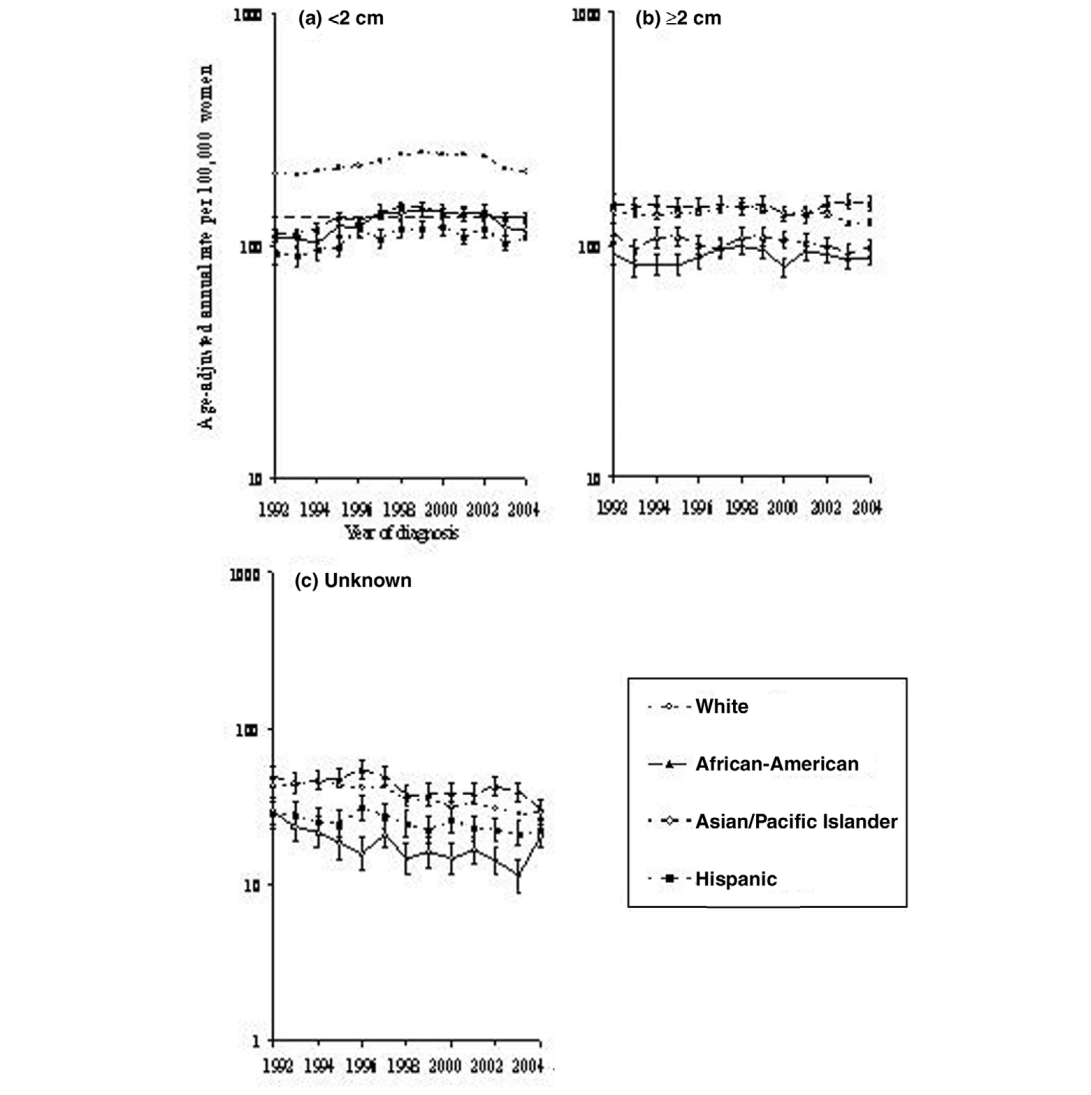
Invasive breast cancer incidence among women 50 years old or older by tumor size, race/ethnicity, and year according to Surveillance, Epidemiology, and End Results-13. **(a) **Trends for tumors with diameter less than 2 cm. **(b) **Trends for tumors with diameter greater than or equal to 2 cm. **(c) **Trends for unknown tumors. All rates are age-adjusted to the 2000 US standard.

Figure 4 shows average annual incidence rates according to breast tumor size by racial/ethnic group. In Asian/Pacific Islander women, incidence of small tumors fell from 136.7 (95% CI 127.5 to 146.4) in 2001 to 117.0 (95% CI 109.1 to 125.4) in 2004, a 14.4% decline comparable to the 16.8% reduction seen in White women (248.2, 95% CI 243.2 to 253.4 in 2001 to 206.6, 95% CI 202.1 to 211.2 in 2004) (Figure 4). The rate of large tumors remained constant over the same period in Asians/Pacific Islanders but dropped 10.2% in Whites. There were no significant changes detected in Hispanics or African-Americans.

Joinpoint regression identified a progressive increase in the incidence rate of small tumors among Asian/Pacific Islander women from 1992 to mid-2000 (1.1% per quarter, 95% CI 0.8% to 1.5%) followed by a decrease from mid-2000 to the end of the observation period (-1.6% per quarter, 95% CI -2.4% to -0.8%). Incidence rates of small tumors in African-Americans and Hispanics also increased initially (1.1% per quarter, 95% CI 0.7% to 1.5%, and 1.0% per quarter, 95% CI 0.6% to 1.4%, respectively) then inflected in mid-1998 and the third quarter of 1999, respectively, to decline more gradually. By comparison, three trends for small tumors were detected in Whites: an increase from 1992 to mid-1999 (0.9% per quarter, 95% CI 0.8% to 1.1%), a nonsignificant decrease from mid-1999 to 2002 (-0.4% per quarter, 95% CI -1.2% to 0.4%), and a sharp decrease after 2002 (-1.8% per quarter, 95% CI -2.4% to -1.2%). Rates of large tumors during 1992–2004 were constant among Asians/Pacific Islanders and African-Americans, decreasing among Hispanics (-0.2%, 95% CI -0.4% to -0.04%), and disjointed among Whites (-0.6% per quarter, 95% CI -1.4% to 1.1% from 1992 to mid-1994; 0.7% per quarter, 95% CI 0.2% to 1.2% from mid-1994 to first quarter 1998; -0.6% per quarter, 95% CI -0.8% to -0.5% from first quarter 1998 to 2004).

## Discussion

Understanding the recent declines in breast cancer incidence requires examination of trends by important mediators of breast cancer heterogeneity, particularly patient race/ethnicity and tumor subtype [1-3,6]. Prior quantifications of breast cancer incidence trends that did not stratify by race/ethnicity may have been biased by changing racial/ethnic composition. In fact, this analysis did find substantial racial/ethnic variation in recent breast cancer incidence trends among women older than 50 at diagnosis, with attenuated declines among Asian/Pacific Islander and Hispanic women and no significant drops among African-American women. For the particular population subgroups with observable reductions, rates of hormone receptor-positive, lobular, and small tumors decreased most markedly. Incidence of *in situ *tumors did not change notably for any non-White group studied.

Racial/ethnic patterns of change speak to the two major hypotheses advanced thus far to explain the recent breast cancer declines: (a) the widespread discontinuation of postmenopausal estrogen/progestin HT in the second half of 2002 [1-3,12-16] and (b) the presumed effects of mammography saturation [2,5]. HT was once the most commonly used drug among US women, with prescriptions peaking at 92 million per year in late 1999 or early 2000, after which growth in prescription rates flattened, presumably due to the release of null results from the Heart and Estrogen/Progestin Replacement Study (or HERS) in 1998, discouraging preliminary reports from the WHI, and restrictive guidelines for HT use disseminated by the American Heart Association [12,27-29]. This plateau in HT prescriptions was observed among all racial/ethnic groups [27]. In July 2002, highly publicized negative findings from the WHI precipitated an immediate and large decline in HT prescriptions, estimated as 37% to 72% in various populations [12-16]. The magnitude of these relative reductions was comparable among Asians/Pacific Islanders, African-Americans, and Whites [30]. A second but not mutually exclusive explanation for observed incidence trends involves population saturation of mammographic screening programs [2,5]. Theoretically, breast cancer incidence, especially rates of small and *in situ *tumors, should plateau or decline once the pool of previously unscreened women is depleted. Recent publications have reported small decreases in mammography uptake between 2000 and 2004 among US women 50 years old or older [31-33].

Overall, our data support a role for mass HT cessation in explaining the incidence patterns described in this analysis. The observation that incidence changes were most pronounced for hormone receptor-positive, lobular, and small tumors corresponds to the limited race/ethnicity-specific data on HT prevalence and discontinuation. Baseline HT use was most prevalent among Whites and progressively less common among Asians/Pacific Islanders, Hispanics, and African-Americans, who were significantly less likely than other racial/ethnic groups to take and continue to use exogenous hormones [30,31,34-39]. California Health Interview Survey data suggest that in 2001 21% of White, 19% of Asian, 13% of Hispanic, and 10% of African-American women older than 50 were current estrogen/progestin users. By 2003, these prevalence estimates were 14% in Whites, 8% in Asians and Hispanics, and a constant 10% in African-Americans [31,40]. Thus, at least in California, absolute changes in HT use were most dramatic among Whites and Asians and least among African-Americans.

Consistent with the evidence that HT acts as a late-stage promoter of hormone-sensitive tumors [41], we detected drops in the rates of ER^+^/PR^+^, but not ER^-^/PR^-^, tumors in Asian/Pacific Islander and White women, the two largest groups of former HT users [12-16]. Increases in the incidence rates of ER^-^/PR^- ^with stable rates of ER^+^/PR^+ ^tumors occurred in the two groups known to use HT less frequently prior to the WHI: Hispanic and African-American women. These groups have been reported to have a higher risk of receptor-negative tumors than White women [19]. Current or recent HT use has also been associated with lobular, ducto-lobular, tubular, tubo-lobular, and, to a lesser extent, ductal tumors [42-46]. While we lacked adequate statistical power to examine trends for these rarer histologic subtypes in non-White groups, the largest declines among Whites occurred for lobular and ducto-lobular breast tumors. Moreover, the substantial decreases in the rates of small tumors among Asians/Pacific Islanders and Whites are in line with evidence that current HT users are more likely to be diagnosed with small but not large tumors when compared with never-users [42]. Although women taking HT may have better access to care and therefore be more likely to receive mammograms, tumor size distributions are reported to be comparable between screened and unscreened HT users Molceular and genetic discov[42]. Molecular and genetic studies indicate that tumors of different sizes may have distinct etiologies, which are established prior to diagnosis [47]. Regardless, since HT appears to preferentially promote growth of hormone-sensitive invasive tumors but not influence risk of *in situ *lesions [11], reductions in the incidence rate of small invasive tumors without substantial changes in the incidence of *in situ *lesions in population subgroups who most used HT are consistent with a major impact of mass HT discontinuation on overall trends.

Our findings do not clearly substantiate the hypothesis that changes in mammographic screening primarily drove the reductions in breast cancer rates. National behavioral risk factor surveys suggest that the percentages of women meeting mammography recommendations leveled off between 1998 and 2000 in all racial/ethnic groups [48,49], with subtle reductions reported between 2000 and 2004 which were larger for Hispanic (-6.2%) than White (-1.5%) women [48]. In California, there was no difference in screening trends by HT use status, with the percentages of women receiving mammograms increasing 12.3% and 11.0% between 2001 and 2003 for HT users and non-users, respectively [31]. Additionally, changes in mammography uptake cannot account for observed drops according to tumor subtype. First, although mammographic screening is more sensitive to receptor-positive than receptor-negative tumors, differences in detection rates are not likely to be substantial enough to explain differential incidence declines by receptor status [50]. Second, mammography is less sensitive to lobular and mixed lobular tumors than ductal tumors because of the more diffuse presentation of the former, so a putative change in screening would have resulted in a larger drop in ductal than lobular tumors, particularly among Hispanics, who had the greatest decline in mammography use, a trend not evident in these data [18,42,50]. Third, reductions in mammographic screening would theoretically minimize lead time so as to reduce the diagnosis of small and *in situ *tumors, which cannot be detected clinically. If mammography saturation caused the majority of changes in breast cancer incidence, we would anticipate equally large declines in small and *in situ *tumors with subtle or no reductions in large tumors. As reported by Jemal and colleagues [2] for US women of all races combined, we found no apparent reductions in the rates of *in situ *cancer for US Asian/Pacific Islander, Hispanic, or African-American women between 2001 and 2004.

Other proposed mechanisms are also unlikely to explain recent breast cancer trends [51]. There has been no evidence of abrupt changes in chemopreventive or other pharmaceuticals (for example, tamoxifen, raloxifene, nonsteroidal anti-inflammatory medications, and statins) relevant to breast cancer between 2000 and 2004 [1,48,51]. And while increased detection of *in situ *tumors over the past two decades could theoretically result in later declines in invasive disease, if *in situ *tumors represent pre-invasive lesions, such a longstanding process is unlikely to account for the dramatic reductions seen over a 2-year period [51]. Thus, for now, widespread discontinuation of HT remains the most plausible explanation for reported declines in breast cancer incidence.

This study used the largest, most extensive population-based cancer database available in the US. SEER registries collect cancer incidence information according to high-quality and rigorous standards [52]. Even though we selected a study period during which highly concordant hormone receptor assays were used to determine ER and PR status [53] and there were no major revisions in diagnostic criteria for histologic categories [18], tumor subtype classification may have differed between pathologists. Similarly, temporal improvements in the reporting of tumor receptor status to cancer registries may have upwardly biased some of the incidence trends reported for receptor-defined subtypes. Thus, the post-2002 declines in ER^+^/PR^+ ^tumors described here and elsewhere may have underestimated the true drop.

Misclassification of patient race/ethnicity is well described in cancer registry data and may have influenced our analyses. Studies investigating the validity of cancer registry race/ethnicity data substantiate excellent overall agreement between SEER and self-reported classifications for non-Hispanic Whites and African-Americans but intermediate agreement for Hispanics and Asians [54,55]. For lack of appropriate population denominators, we were unable to investigate trends among Asian subgroups, which have previously been shown to have differing incidence patterns [56]. Another limitation of this analysis was its low statistical power to detect subtle trends in particular breast cancer subtypes among Asians/Pacific Islanders and Hispanics. We could not evaluate rates for tubular breast cancer because of its low yearly incidence, nor could we examine additional breast cancer subtypes such as luminal A, luminal B, or HER2/neu-defined tumors because SEER does not yet report these characteristics.

## Conclusion

Reductions in postmenopausal breast cancer incidence observed in the US were strongest in White women, intermediate in Asian/Pacific Islander and Hispanic women, and absent in African-American women. In all groups, declines were most evident for hormone receptor-positive, lobular, and small tumors. While this and other ecologic analyses cannot definitively speak to the influence of population-wide reductions in mammographic screening or other risk factors, disproportionate declines in the incidence of hormone-sensitive tumor subtypes among racial/ethnic populations that most commonly used HT bolster the hypothesis that mass HT discontinuation after mid-2002 was the predominant cause of recent breast cancer declines. To further inform discussions about the impact of population-level HT use on breast cancer, cohort studies, including the WHI observational cohort, should examine the relationship between timing of HT cessation (and possible resumption) and risk of specific breast cancer subtypes. Future surveillance of population-based breast cancer trends must stratify findings by racial/ethnic group and tumor subtypes.

## Abbreviations

CI = confidence interval; ER = estrogen receptor; HT = hormone therapy; ICD-O-3 = *International Classification of Diseases for Oncology, 3rd Edition*; IDC = invasive ductal carcinoma; IDLC = invasive ducto-lobular carcinoma; ILC = invasive lobular carcinoma; PR = progesterone receptor; SEER = Surveillance, Epidemiology, and End Results; WHI = Women's Health Initiative.

## Competing interests

CAC has served as an expert witness for the plaintiffs in hormone therapy litigation. The other authors declare that they have no competing interests.

## Authors' contributions

AKH and CAC conceived of the study, performed the statistical analysis, interpreted the data, and drafted the manuscript. THK and ETC participated in critical revisions of the manuscript. All authors read and approved the final manuscript.
